# Cu/Ce-co-Doped Silica Glass as Radioluminescent Material for Ionizing Radiation Dosimetry

**DOI:** 10.3390/ma13112611

**Published:** 2020-06-08

**Authors:** Jessica Bahout, Youcef Ouerdane, Hicham El Hamzaoui, Géraud Bouwmans, Mohamed Bouazaoui, Andy Cassez, Karen Baudelle, Rémi Habert, Adriana Morana, Aziz Boukenter, Sylvain Girard, Bruno Capoen

**Affiliations:** 1 Univ-Lille, CNRS, UMR 8523-PhLAM-Physique des Lasers Atomes et Molécules, F-59000 Lille, France; jessica.bahout.etu@univ-lille.fr (J.B.); hicham.el-hamzaoui@univ-lille.fr (H.E.H.); geraud.bouwmans@univ-lille.fr (G.B.); mohamed.bouazaoui@univ-lille.fr (M.B.); andy.cassez@univ-lille.fr (A.C.); karen.baudelle@univ-lille.fr (K.B.); remi.habert@univ-lille.fr (R.H.); 2 Univ Lyon, Laboratoire H. Curien, UJM-CNRS-IOGS, 18 rue du Pr. Benoît Lauras 42000, Saint-Etienne, France; ouerdane@univ-st-etienne.fr (Y.O.); adriana.morana@univ-st-etienne.fr (A.M.); aziz.boukenter@univ-st-etienne.fr (A.B.); sylvain.girard@univ-st-etienne.fr (S.G.)

**Keywords:** Cu^+^/Ce^3+^-co-doped silica glass, photoluminescence, ionizing radiation, radioluminescence, dosimetry

## Abstract

Optically activated glasses are essential to the development of new radiation detection systems. In this study, a bulk glassy rod co-doped with Cu and Ce ions, was prepared via the sol-gel technique and was drawn at about 2000 °C into a cylindrical capillary rod to evaluate its optical and radioluminescence properties. The sample showed optical absorption and photoluminescence (PL) bands attributed to Cu^+^ and Ce^3+^ ions. The presence of these two ions inside the host silica glass matrix was also confirmed using PL kinetics measurements. The X-ray dose rate was remotely monitored via the radioluminescence (RL) signal emitted by the Cu/Ce scintillating sensor. In order to transport the optical signal from the irradiation zone to the detection located in the instrumentation zone, an optical transport fiber was spliced to the sample under test. This RL signal exhibited a linear behavior regarding the dose rate in the range at least between 1.1 mGy(SiO_2_)/s and 34 Gy(SiO_2_)/s. In addition, a spectroscopic analysis of this RL signal at different dose rates revealed that the same energy levels attributed to Cu^+^ and Ce^3+^ ions are involved in both the RL mechanism and the PL phenomenon. Moreover, integrated intensities of the RL sub-bands related to both Cu^+^ and Ce^3+^ ions depend linearly on the dose rate at least in the investigated range from 102 mGy(SiO_2_)/s up to 4725 mGy(SiO_2_)/s. The presence of Ce^3+^ ions also reduces the formation of HC1 color centers after X-ray irradiation.

## 1. Introduction

The development of new radiation detection systems based on doped host glasses is the subject of intense investigations [1,2,3,4]. Both single Ce^3+^- and Cu^+^- activated silica glasses constitute promising candidates for ionizing radiation dosimetry with configurations that allow real-time and/or remote measurements [5,6]. Indeed, sol-gel-derived Ce^3+^- or Cu^+^- doped glasses excited by UV lasers or X-rays exhibit emission signals in blue and green regions, respectively [7,8]. Based on these optical activities and on the ability to control the size and shape of sol-gel samples, efficient copper or cerium-doped silica glasses were used as scintillators in distant dosimeters coupled with optical fibers. Those materials dedicated to dosimetry were sensitive to X-rays thanks to radioluminescence (RL) and optically stimulated luminescence (OSL) processes [7,8,9,10]. Monovalent copper ion (Cu^+^) is particularly interesting as a dopant for sol-gel silica because it exhibits strong luminescence quantum yields in the visible region if the xerogel is densified in neutral atmosphere [11]. In the case of copper-doped silica glasses, however, compared to cerium-doped ones, a darkening effect appeared after exposure to a dose level of 50 kGy(SiO_2_), although the material remained X-ray-sensitive [8]. This effect is caused by the appearance of radio-induced colored centers, such as HC1, presenting visible absorption bands [12]. In addition, we have shown that silica glass doped with cerium ions shows a higher resistance to X-rays in comparison with a Cu^+^-activated sample [7]. This is in agreement with previous research works demonstrating the ability of Ce-doping to reduce the formation of radio-induced colored centers in silicate glasses [13,14,15]. Hence, to benefit from the Cu-doped silica scintillator performances without the disadvantage of its darkening, a copper-cerium co-doping is investigated as an enhanced solution. Moreover, the presence of two kinds of luminescent centers should extend the luminescence spectral range, thereafter, enhancing the detection capabilities by matching the sensitive wavelength region of photon detectors. In particular, the green luminescence of Cu^+^ ions would be preferred for dosimetry of MeV-range radiation because in this case, the Cerenkov stem effect has a maximal contribution at shorter wavelengths, and it can be more easily separated from the RL signal. Thus, in this work, Cu/Ce-co-doped silica glass was synthesized via a sol-gel route to evaluate its optical properties (absorption, photoluminescence) and RL response under X-rays.

## 2. Materials and Methods

### 2.1. Glass Fabrication

Silica xerogel was synthetized, at FiberTech Lille (Lille, France) platform, using tetraethyl orthosilicate (TEOS) as a precursor, like it has already been described in [16]. The obtained porous xerogel, once stabilized in a furnace at 1000 °C, was soaked into a solution of cerium and copper salts dissolved in ethanol. After this, the silica monolith was taken out of the solution and put in an oven at 50 °C for 24 h in order to remove solvents, to retain Ce and Cu dopants inside the matrix. The obtained doped porous silica was then heat-treated for 2 h at 1200 °C in helium gas for its densification. Finally, the Cu/Ce-co-doped glassy rod was drawn down to millimeter-sized canes by stretching it at about 2000 °C. The Cu and Ce concentrations were measured (by Electron Probe Micro Analysis) in single-doped materials [9,11]. They were estimated to 250 and 300 atomic ppm for Cu and Ce, respectively. The experimental procedures to produce, then to dope and to densify the initial xerogels are similar in both single-doped and co-doped samples. Hence, with the same concentrations of doping solutions, we expect the final dopant concentrations to be similar.

### 2.2. Characterizations

Room temperature absorption spectroscopy in the UV-Visible range was carried out with a Cary 5000 spectrophotometer (Agilent, Santa Clara, CA, USA).

For time-resolved luminescence (TRL) experiments, the excitation laser source was based on an optical parametric oscillator equipped with a second harmonic generation nonlinear crystal pumped by the third harmonic of a Nd:YAG laser. The selected probe signal was characterized by a pulse width duration of 5 ns and a repetition rate of 10 Hz. The light emitted by the samples was spectrally resolved using a grating-based spectrometer with 300 grooves/mm and it was then recorded by a sensitive gated intensified CCD (PI-MAX high speed gated camera, Princeton Instruments, Trenton, NJ, USA) equipped with a time window-delay generator.

**Figure 1:**
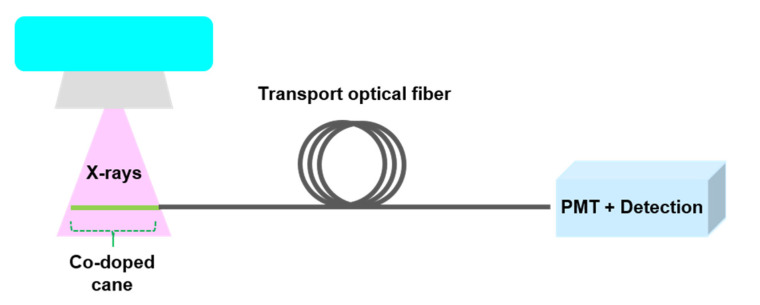
Measurement setup of the employed Radio Luminescence (RL)-based dosimeter.

RL measurements were performed by using the setup shown in Figure 1. A piece of the Cu^+^/Ce^3+^-co-doped silica cane, with diameter 0.5 mm and length 1 cm, was welded to the tip of a multimode pure silica fiber having a length of about 5 m. Such passive fiber elaborated with a 0.5 mm pure silica core size diameter while a low refractive index polymer was used for the cladding part. This fiber was used to collect the RL signal and to waveguide it towards a photomultiplier module (PMT, H9305-03 Hamamatsu, Hamamatsu, Japan). The amplified voltage at the PMT level was proportional to the optical signal amplitude emitted by the Cu/Ce-co-doped sample under test. For data analysis, the output analog PMT signal was recorded with a numerical oscilloscope (InfiniiVision DSO7052B, Agilent Technologies, Santa Clara, CA, USA). It should be noted that since the PMT integrates the whole emission spectrum, the obtained signal comes from all the emitting centers, without any ion spectral signal selection. For RL measurements, X-ray irradiations were performed with the MOPERIX facility at Laboratoire Hubert Curien (Saint Etienne, France). The machine was set at a high voltage of 100 kV and delivered photons with a mean energy of ~40 keV. The dose rate was monitored by either the electric current of the MOPERIX machine or by the controlled distance between the X-ray source and the samples under test. A calibrated ionization chamber was used to evaluate the dose rates in Gy(H_2_O)/s. Then, the dose rates were expressed in Gy(SiO_2_) by applying a factor that takes into account the mass attenuation coefficients of water and silica.

RL spectra were collected by exchanging the PMT detector for a UV/VIS mini-spectrometer (C10082CA Hamamatsu, Hamamatsu, Japan) in Figure 1. For each RL spectrum, the dark signal, due to the device, was subtracted before analysis. Furthermore, the reported room-temperature PL spectra were recorded using the same pulsed laser source as for the TRL measurement. The emitted light was then collected through the connection of the fiber to the same Hamamatsu UV/VIS mini-spectrometer, in order to accurately compare RL and PL spectra.

## 3. Results and Discussion

### 3.1. Absorption and Photoluminescence

**Figure 2:**
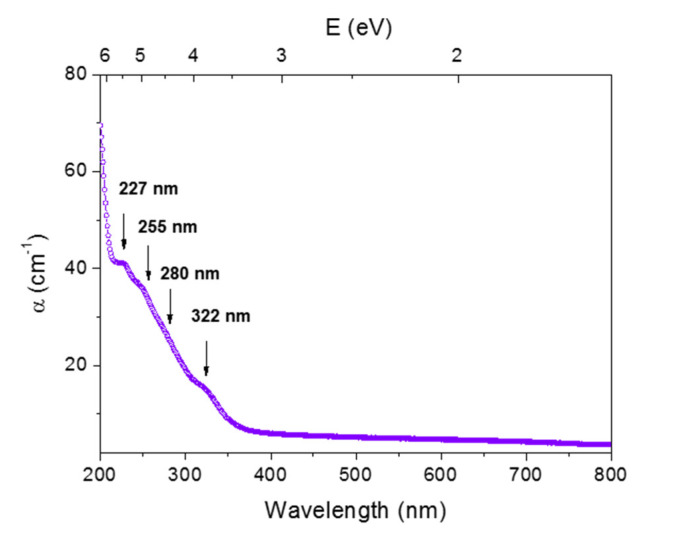
UV-Visible optical absorption spectra of Cu/Ce-co-doped silica cane.

Figure 2 shows the optical absorption spectrum of the Cu/Ce-co-doped silica cane, presenting a rather broad band in the 200–375 nm spectral range, constituted of four main absorption peaks around 227, 255, 280 and 322 nm. Some of these peaks can only be guessed, as shoulders, in the spectrum because they are superimposed to the tail of a larger band situated in the deep UV and caused by defects associated with the SiO_2_ host silica matrix. Based on the results of our previous studies related to Ce^3+^ or Cu^+^ single doping, some of these absorption bands can be attributed: for example, it is shown in [11] that Cu^+^ ions have a large absorption band peaking around 296 nm in a copper-doped silica glass prepared using the same technique. This band at 296 nm cannot be detected in the spectrum of Figure 2, but instead, a shoulder can be observed around 280 nm, which can be reasonably ascribed to 3d^10^→3d^9^4s transition of Cu^+^. Indeed, the two positions (280 and 296 nm) are not far apart, being given the band width and the uncertainty of its position that strongly depends on the ion environment [17,18,19]. The band centered at 255 nm appears close to the maximum at 260 nm of a large band ascribed to charge transfer transitions from ligands to Ce^4+^ cations in pure silica [20,21]. In our reference [9], the corresponding maximum was found at 258 nm and the band width is of about 80 nm. Finally, the small peak at 322 nm can be assigned to 4f→5d transition of Ce^3+^ ions [9,20]. As for the small peak around 227 nm, it has never been observed neither in Cu-doped glasses nor in Ce-doped ones. Thus, it should be ascribed to a Ce/Cu co-doping effect and its impact on the structure of the host glass matrix. The presence of a similar absorption band was observed in silica glasses co-doped with Yb/Al [22].

**Figure 3:**
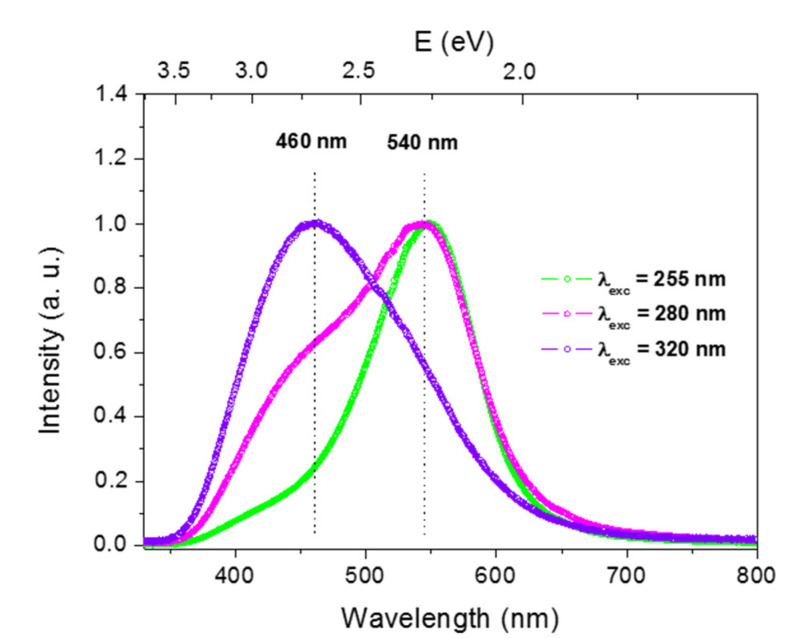
Normalized PL spectra of Cu/Ce-co-doped cane under 255 nm, 280 nm and 320 nm laser excitation.

Normalized PL spectra of the Cu/Ce-co-doped cane under different laser excitations are presented in Figure 3. The excitation wavelengths roughly correspond to the three absorption bands identified in Figure 2 and associated to ionic cerium or copper. It can be seen that the obtained spectra exhibit broad bands roughly consisting of two individual peaks centered around 460 and 540 nm, respectively. The relative intensities of these two bands strongly depend on the excitation wavelength. For instance, under excitation at 320 nm, the emission intensity around 460 nm is higher compared to the one around 540 nm. On the contrary, when the sample is excited at 280 nm, the signal intensity around 540 nm becomes higher than that around 460 nm, although both bands remain in the same order of magnitude. Finally, under 255 nm excitation, the green luminescence becomes overwhelming. The luminescence bands around 460 nm and 540 nm are attributed, according to the literature [7,19,23,24], to Ce^3+^ and Cu^+^ ions, respectively. Hence, the maximum of the luminescence band can be tailored by choosing the excitation: Ce^3+^ ions alone, a mix of Ce^3+^ and Cu^+^ ions or Cu^+^ ions alone can be preferentially excited at 320 nm, 280 nm or 255 nm, respectively. In fact, the dissymmetry of the PL bands in Figure 3 is caused by the contribution of five energy levels, two of them being related to Cu^+^ ions, two others associated with Ce^3+^ ions and one last level due to non-bridging oxygen hole centers (NBOHC) point defects, as explained in more details in Section 3.2.

To confirm that the blue part of the emission spectrum corresponds to the emission by Ce^3+^ ions, while the green part corresponds to emission by Cu^+^ ions, PL kinetics measurements were performed under UV excitations. The objective of these measurements is to confirm the emission attributions by exciting the material with the right excitations and by detecting the signal at the wavelengths associated with transitions between levels in the two ions, which have specific and different lifetime ranges.

**Figure 4:**
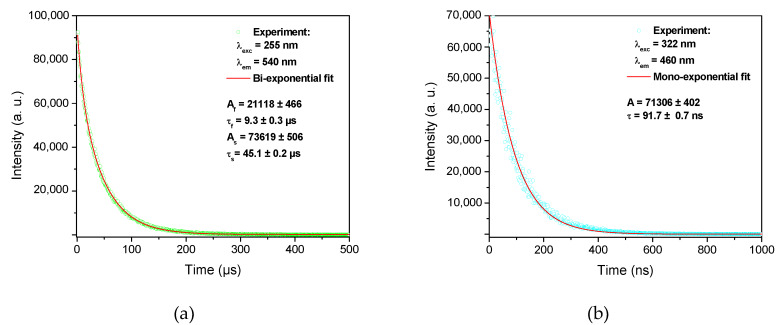
PL decay curves of Cu/Ce-co-doped cane at different emission wavelengths: (**a**) 540 nm with 255 nm-excitation and (**b**) 460 nm with 322 nm-excitation.

Figure 4 shows PL decay curves of the co-doped sample recorded at 540 nm and 460 nm upon excitations at 255 nm and 322 nm, respectively. The signal at 540 nm (Figure 4a) has a two-exponential behavior with a fast and a slow component, assigned to decay times τ_f_ = 9.3 ± 0.3 µs and τ_s_ = 45.1 ± 0.2 µs, respectively. The obtained values are characteristic of Cu^+^ ions. Attributed to transitions in a three levels model, they are similar to the decay times measured in silica glasses doped with only Cu ions [12,19]. At 460 nm (Figure 4b), PL decay curves behave like mono-exponential functions, with a characteristic time τ = 91.7 ± 0.7 ns. Such a time, of several tens of nanoseconds, is typical for measured PL lifetimes of Ce^3+^ ions in various silica-based glasses [20,24,25].

### 3.2. Radioluminescence

It is worthy to recall that the aim of the Cu/Ce co-doping is to take advantage of the high efficiency of both Cu^+^ and Ce^3+^ ions, to enlarge the emission spectra and protect the silica matrix from irradiation-induced defects by using cerium ions.

**Figure 5:**
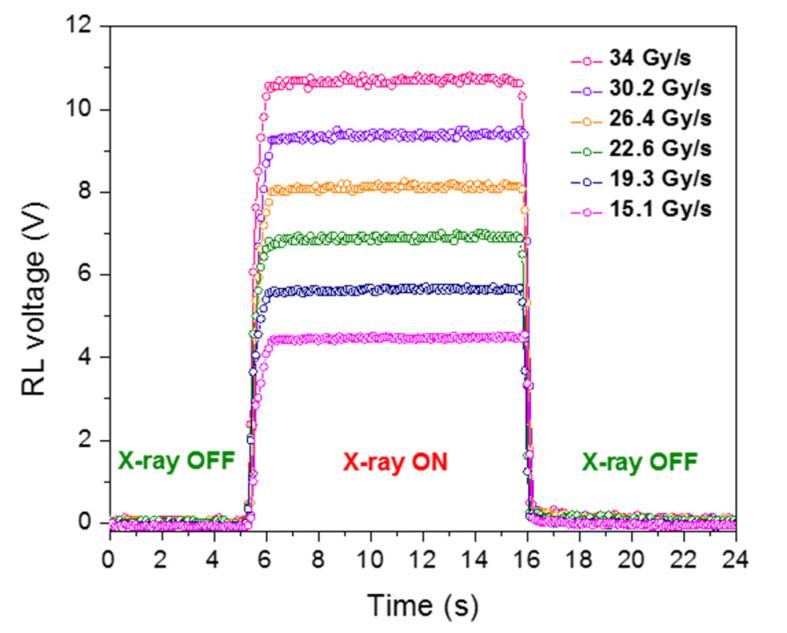
RL signal of the Cu/Ce-co-doped cane spliced to a transport fiber versus time for different dose rates.

The RL signal of the Cu/Ce-co-doped glass, recorded using the PMT module is shown in Figure 5. When comparing the curves of this figure to the one related to a Cu^+^-doped rod [8], they all have the same general trend. However, it cannot be said that Ce^3+^ ions have a beneficial role in the RL intensity level because we are not able to perform a quantitative comparison, as the setups may be slightly different in the two experiments. At the beginning of the irradiation, the RL signal starts to increase for about one second before reaching its permanent regime. This delay in the material response is attributed to carrier relaxation through shallow trap states. In effect, during irradiation, the prompt recombination causing the production of the RL response competes with carriers trapping by shallow and deep defect levels. Nevertheless, as the carrier traps are being filled, the trapping probability drops, leaving more available carriers for radiative recombinations. The RL signal then reaches its steady-state, the magnitude of which increases with dose rate [7]. A stable plateau and no long decay time afterglow are noticeable key characteristics of the RL signal from this Cu/Ce-co-doped cane. This can be explained by a rather reduced number of shallow traps and/or by a short-time access to the steady state equilibrium between carrier-trapping and detrapping rates during irradiation.

**Figure 6:**
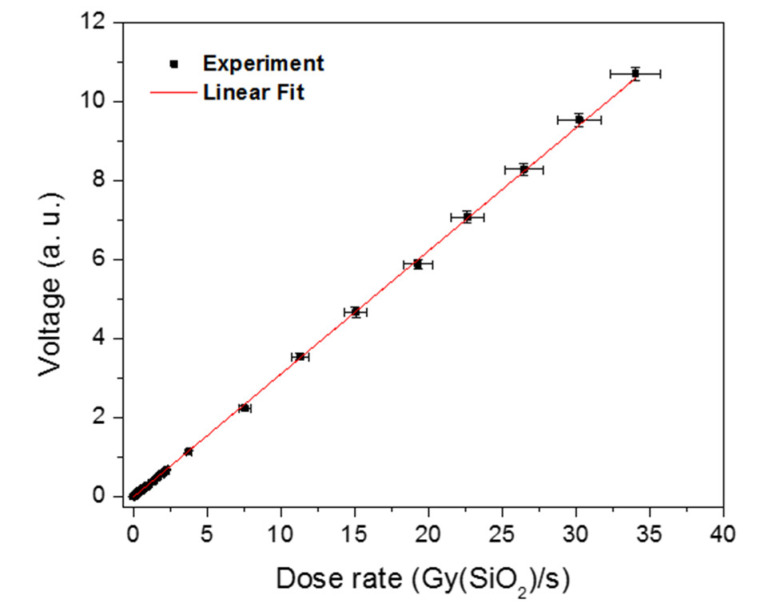
RL signal amplitude of the Cu/Ce-co-doped sample in permanent regime reported versus the X-ray dose rate.

The reproducibility of the RL intensities, for each dose rate, was checked by several measurements and was estimated to 2%. This reproducibility was unchanged after at least several kGy of accumulated dose. Indeed, the stability and robustness of the sensor were tested and verified for several runs and for several kGy of cumulative doses. Figure 6 reports the stable signal amplitude evolution versus the dose rate, showing linearity with the dose rate in the minimal range between 1.1 mGy(SiO_2_)/s and 34 Gy(SiO_2_)/s. Consequently, despite the highest dose rate accessible with our irradiation facility (of 34 Gy(SiO_2_)/s), the linear trend previously obtained with single Cu- or Ce- doped silica glasses [9,10] (up to 30 Gy(SiO_2_)/s) could be confirmed and even overtaken with Cu/Ce-co-doping.

**Figure 7:**
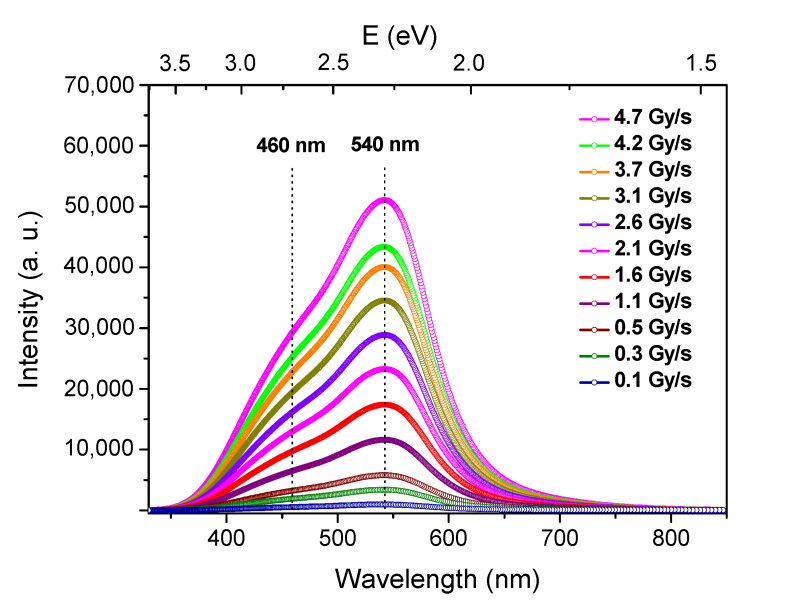
RL spectra of the Cu/Ce-co-doped cane spliced to a transport fiber obtained at different X-ray dose rates.

**Figure 8:**
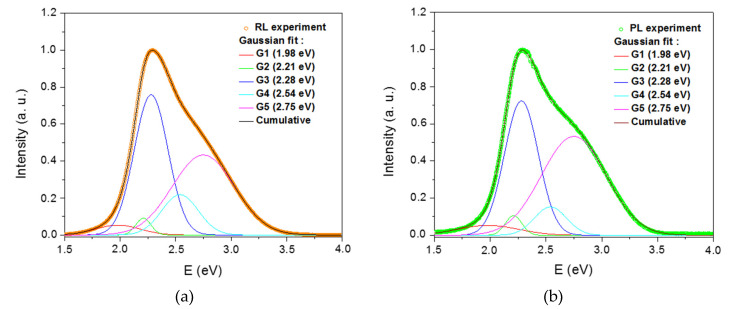
Gaussian shape decomposition of the (**a**) RL spectrum and (**b**) PL spectrum of Cu/Ce-co-doped cane obtained under X-irradiation (4.7 Gy(SiO_2_)/s) and UV laser excitation (280 nm), respectively.

We also carried out spectroscopic measurements to clarify the origins of the RL process. Figure 7 shows the RL spectrum of the Cu/Ce-co-doped glass under different X-ray dose rates. At first sight, the broad emission spectra consist of two main bands around 460 and 540 nm, respectively, as was the case for the PL spectra (Figure 3). Moreover, the overall intensity of this RL emission increases with the dose rate, while its shape remains similar. In fact, the dissymmetrical shape of this emission spectra is well fitted to five Gaussian bands centered at 1.98 eV (626 nm; G1), 2.21 eV (561 nm; G2), 2.28 eV (543 nm; G3), 2.54 eV (488 nm; G4) and 2.75 eV (451 nm, G5) (Figure 8a). The two bands peaking at 561 nm and 543 nm can be assigned to the emission from ^3^Eg” levels of Cu^+^ ions, i.e., from monovalent ions in octahedral symmetry with tetragonal distortion [8,19]. The bands at 488 nm and 451 nm are assigned to the transition from 5d to 4f states of Ce^3+^, the ground level 4f being split by spin-orbit interaction into sublevels ^2^F_5/2_ and ^2^F_7/2_ [7,26]. The last band at 626 nm can be assigned to NBOHC silica defect [27,28]. Such NBOHCs are presumed to be caused by the rod drawing conditions [29,30].

Since the PL spectra recorded under 280 nm laser excitation present a very similar shape as that of the RL spectra (Figure 8), this proves that the same energy levels attributed to Cu^+^ and Ce^3+^ ions are involved in both the RL mechanism and the PL phenomenon. More precisely, in both spectroscopies, the end transitions occur mainly between excited and ground states of both Ce^3+^ and Cu^+^ activating ions. The NBOHC silica defects also contribute to the RL and PL processes.

As in the case of RL spectrum (Figure 8a), a PL spectrum (Figure 8b) was fitted using five Gaussian bands with the same spectral positions. Based on the obtained Gaussian sub-band areas (Table 1), in the case of RL spectrum, the contributions of Cu^+^ ions (G2 + G3), Ce^3+^ ions (G4 + G5) and NBOHCs (G1) to the total RL signal were of about 42.05%, 54.3% and 3.65%, respectively. Concerning the PL spectrum, the contributions were of about 38.08%, 57.19% and 4.73% for Cu^+^ ions, Ce^3+^ ions and NBOHCs, respectively. The weak differences between the PL bands and their RL counterparts are supposed to be due to different mechanisms of excitation and emission between the two processes. Indeed, in the case of the PL process, incident photons have more or less the right energy to directly excite the Ce^3+^ and Cu^+^ ions. On the contrary, in the case of the RL process, the X-ray irradiation first initiates electron-hole pairs creation. Then, these carriers need to relax through defect levels and finally recombine radiatively on the ion centers.

**Figure 9:**
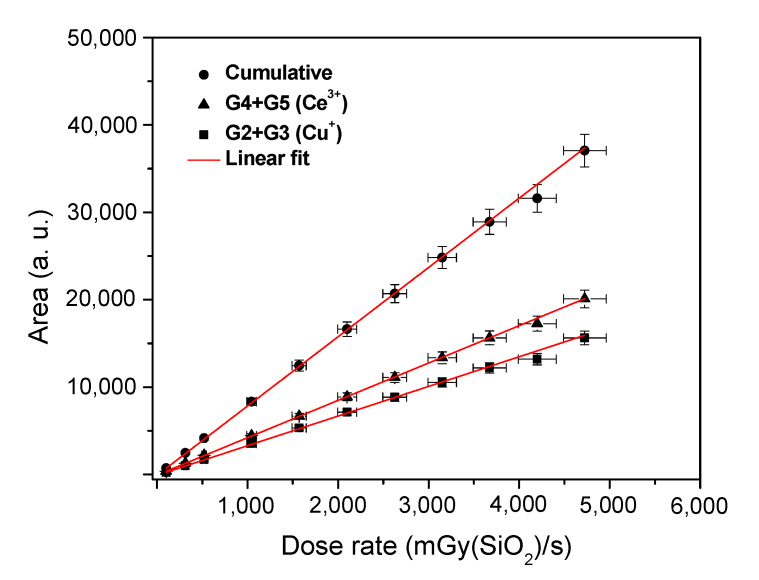
Integrated intensities of the whole RL spectrum and of Gaussian sub-bands related to Cu^+^ (G2 + G3) and Ce^3+^ (G4 + G5) ions plotted versus the dose rate.

Figure 9 shows the evolution of the total integrated RL intensity taken from the spectra of Figure 7 upon the dose rate, together with the integrated RL intensities of Gaussian sub-bands related to Cu^+^ and Ce^3+^ ions, as it was calculated from Figure 8a and reported in Table 1. Independently of the active ion, the plots remain fully linear at least in the investigated dose rate range, namely from 102 mGy(SiO_2_)/s up to 4725 mGy(SiO_2_)/s.

### 3.3. Radio-Darkening

**Figure 10:**
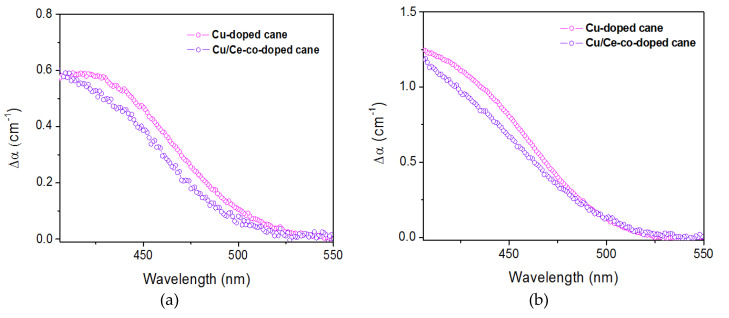
Radiation induced attenuation spectra after X-irradiation at (**a**) 50 kGy(SiO_2_) and (**b**) 1 MGy(SiO_2_) on Cu-doped and Cu/Ce-co-doped canes.

In our previous study related to single Cu-doped sol-gel bulk glasses, we reported an ionizing radiation-induced absorption in the blue spectral range. This optical absorption was assigned to holes trapped on nonbridging oxygens of HC1-type [12]. Such absorption could be detrimental to the performance of sensors based on visible luminescence. In our co-doped sample, the presence of Ce^3+^ ions could have an anti-darkening effect due to their ability to trap holes [31]. Hence, the effect of X-irradiation on optical absorption of both Cu/Ce-co-doped and Cu single doped drawn canes, made using the same technique in similar conditions, was determined after an X-ray irradiation at doses of 50 kGy(SiO_2_) and 1 MGy(SiO_2_). Figure 10 shows the X-irradiation induced optical absorption spectra of Cu-doped and Cu/Ce-co-doped silica samples in the spectral zone characteristic of HC1 defects. Each spectrum was calculated as the difference between the spectra of the sample before and after X exposure. Exposure of both Cu-doped and Cu/Ce-co-doped samples to X-rays results in the appearance of a broad band centered at about 435 nm, which is assigned to HC1 defect centers in silica. However, for both irradiation doses, the level of this absorption band is slightly lower in the case of the co-doped sample. This decrease shows that co-doping the matrix with Ce^3+^ ions mitigates the formation of HC1 color centers by trapping released holes under X-irradiation.

## 4. Conclusions

Cu/Ce-co-doped silica glass was synthesized using a sol–gel protocol. Once drawn into a cane (capillary rod), the sample exhibited the characteristic optical absorption and luminescence spectra attributed to Cu^+^ and Ce^3+^ in SiO_2_ matrix. Such a material showed a RL activity in the visible domain, which can be used for measuring X-ray dose rates. To this purpose, a 1-cm-long sample of this cane, showing the scintillating properties, was welded to a transport passive optical fiber. The obtained device was likely to allow the remote estimation of the X-ray dose rate. The RL response exhibited linearity against the dose rate in a range at least from 1.1 mGy(SiO_2_)/s to 34 Gy(SiO_2_)/s. Spectroscopic investigations demonstrated that identical energy levels—attributed to both Ce^3+^ and Cu^+^ activating ions—were mainly involved in the final steps of the RL phenomenon, as well as in the PL phenomenon. Moreover, the total integrated RL intensity, as well as the integrated RL intensities of Gaussian bands related to Cu^+^ and Ce^3+^ ions, all depended linearly upon the dose rate in the investigated range from 102 mGy(SiO_2_)/s up to at least 4725 mGy(SiO_2_)/s. Finally, we showed that the presence of Ce^3+^ ions had an anti-darkening effect limiting the formation of HC1 color centers. Given these encouraging results, this Cu/Ce-co-doped glass offers a high potential that could be exploited in practical radiation dosimetry.

## Figures and Tables

**Table 1:** Gaussian sub-bands normalized areas (in %) of RL spectra (X-irradiation, 4.7 Gy(SiO_2_)/s)) and photoluminescence (PL) spectra (UV excitation at 280 nm).

Gaussian Band	RL	PL
G1 (NBOHC)	3.65	4.73
G2 (Cu^+^)	2.13	2.55
G3 (Cu^+^)	39.92	35.53
G4 (Ce^3+^)	12.40	7.15
G5 (Ce^3+^)	41.90	50.04
